# Malignant Infantile Osteopetrosis With Neurological and Hematological Complications: A Case Review

**DOI:** 10.7759/cureus.85521

**Published:** 2025-06-07

**Authors:** Tuqa A Abdulsalam, Mira Elmiaari, Layla D ALRomithi, Elham O Al-Fakih, Fatma J Alzarooni

**Affiliations:** 1 Pediatrics, Al Jalila Children’s Hospital, Dubai, ARE

**Keywords:** autosomal recessive bone disorder, bone marrow failure, hematopoietic stem cell transplantation, malignant infantile osteopetrosis, neurological complications, optic nerve atrophy, ostm1 mutation, pathologic fractures

## Abstract

Infantile malignant osteopetrosis is a rare disease characterized by autosomal recessive skeletal dysplasia secondary to defective bone resorption as a consequence of dysfunctional osteoclasts. It manifests in infancy with bone marrow failure, central nervous system disease, and skeletal impairment. Some genetic subtypes may be potentially curable with hematopoietic stem cell transplantation, but the results are overall poor in patients with advanced neurologic involvement or adverse genetic mutations. We describe the case of a six-week-old male infant born to consanguineous parents, who presented with fever, difficulty in breathing, and progressive irritability. On clinical examination, he had coarse facial features, a systolic heart murmur, sacral hair tuft, and dental anomalies. Laboratory workup revealed a severe anemia, thrombocytopenia, and mildly elevated liver enzymes. Despite antimicrobial empiric therapy, the patient's clinical condition worsened, and a radiological examination showed several pathological fractures and increased bone density. Skeletal survey documented generalized bone sclerosis and remodeling, and the case was diagnosed as osteopetrosis. Cerebral magnetic resonance imaging demonstrated a cystic hygroma, and ocular investigation confirmed that both optic nerves were atrophied and visual evoked potentials were absent. Vitamin D deficiency, an elevated parathyroid hormone (PTH), and normal serum calcium were noted on bone and metabolic panels. Whole-exome sequencing revealed malignant infantile osteopetrosis based on a homozygous deletion in the *OSTM1* gene. Immediate care of the infant included the administration of blood and platelet transfusions, nutritional supplementation, and orthopedic intervention. Even though the patient was being scheduled for allogenic haematopoietic stem cell transplantation in a foreign country, he died before the procedure could be arranged. This case underscores the diagnostic challenges and extreme clinical severity of malignant infantile osteopetrosis, especially the genetic subtypes that do not respond to definitive therapies. The mutation in the *OSTM1* gene results in severe neurological decline and a very poor outcome. An early genetic diagnosis, a multidisciplinary approach, and regional genetic studies are essential to improve prognosis in the affected infants. Allogeneic hematopoietic stem cell transplantation provides the only curative therapy for some subtypes, but the prognosis is dismal in the presence of extensive neuraxial disease. This case also highlights the need for early prevention of symptoms, genetic counseling, and the ongoing search for new therapies.

## Introduction

Osteopetrosis is a heterogeneous hereditary bone disorder caused by a reduction in osteoclastic activity with increased bone density. The disease presents with clinical and genetic heterogeneity, it is usually divided into three clinical forms based on its severity and pattern of inheritance: autosomal recessive, malignant infantile osteopetrosis, intermediate autosomal recessive osteopetrosis, and autosomal dominant, adult-onset osteopetrosis [1,2]. The most severe form is the malignant infantile type, which presents in infancy and is associated with haematologic failure, pathologic fractures, and neurological dysfunction secondary to compression of cranial nerves [1,3]. The pathophysiology of malignant infantile osteopetrosis is a failure on the part of osteoclasts to reabsorb bone, leading to obliteration of marrow cavities, and subsequently to anemia, thrombocytopenia, hepatosplenomegaly from extramedullary hematopoiesis, and infections [2,4].

Radiographic findings usually show diffuse osteosclerosis, “bone-in-bone” appearance, and metaphyseal flares of long bones [1,5]. Cranial nerve compression, such as that seen in optic atrophy and sensorineural hearing loss, is frequently secondary to narrowed intracranial foramina [2,5].

Multiple gene mutations have been implicated in the disease; *TCIRG1*, *CLCN7*, and *OSTM1* mutations cause disruption of osteoclast function or differentiation. The *OSTM1* mutation is associated with a severe phenotype with progressive neurological degeneration, optic atrophy, and developmental delay [6]. Although hematopoietic stem cell transplantation can be a curative treatment in some genetic subtypes when done early on, its efficacy is low in patients with *OSTM1* mutations because of poor neurological outcome [6,7].

Malignant infantile osteopetrosis is an extremely uncommon disorder and is thought to affect approximately one in 250,000 of the worldwide population [1]. This case highlights the severe but rapid nature of clinical deterioration associated with this genetic subtype and the importance of early diagnosis, genetic counseling, and multi-disciplinary care.

## Case presentation

Patient background

A six-week-old male term infant delivered vaginally (38 weeks gestational age) presented with fever and increased work of breathing for two days. He also had recurrent episodes of back arching over the past two weeks, not associated with feeding. He was the third child of first-degree cousins, having two older healthy daughters. He had a normal newborn screening, but had a history of neonatal intensive care unit (NICU) admission for severe respiratory distress with three days of non-invasive respiratory support.

Initial presentation

At presentation, the child had mild coarse facial features, which were dismissed initially as family likeness. Other findings were a grade 2/6 apical systolic murmur, sacral hair tuft, Mongolian spot, and a dental cyst, all in the absence of hepatosplenomegaly. He underwent a sepsis workup, including multiple attempts at the lumbar puncture, which resulted in dry tap. A chest radiograph was performed as part of the sepsis workup and revealed clear lung fields without focal consolidation, effusion, or signs of pulmonary pathology. No abnormal comment was noted regarding the ribs or bony thoracic structures.

His laboratory results were significant for the following: white blood cell count 21.2 × 10⁹/L (neutrophils 80%, lymphocytes <40%), hemoglobin 8.3 g/dL, platelet count 75 × 10⁹/L, absolute lymphocyte count 14.56 × 10⁹/L, and absolute neutrophil count 2.92 × 10⁹/L (Table 1). He had mild liver function derangement (alanine transaminase (ALT) 67 IU/L, aspartate aminotransferase (AST) 265 IU/L, alkaline phosphatase (ALP) 433 IU/L) (Table 2). The infant was then commenced on empirical IV ampicillin and cefotaxime for suspected neonatal sepsis.

**Table 1:** Complete blood count at admission MCV: mean corpuscular volume; MCHC: mean corpuscular haemoglobin concentration; RDW: red cell distribution width

Component	Patient Value	Reference Range	Unit
WBC Count	21.15	5.0 – 13.0	×10⁹/L
RBC Count	2.71	4.00 – 5.20	×10¹²/L
Hemoglobin	8.3	11.5 – 15.5	g/dL
Hematocrit	27.2	35.0 – 45.0	%
MCV	100	77.0 – 95.0	fL
MCH	30.6	25.0 – 29.0	pg
MCHC	30.5	31.5 – 34.5	g/dL
RDW	17.7	11.5 – 14.0	%
Platelet Count	75	170 – 450	×10⁹/L
Absolute Lymphocyte Count	14.56	1.5 – 7.0	×10⁹/L
Absolute Neutrophil Count	2.92	1.5 – 7.5	×10⁹/L

**Table 2:** Liver and kidney function tests AST: aspartate aminotransferase; ALT: alanine transaminase; ALP: alkaline phosphatase; SGOT: serum glutamic-oxaloacetic transaminase; SGPT: serum glutamic pyruvic transaminase

Component	Result	Reference Range	Unit
Sodium	143	136 – 145	mmol/L
Potassium	4.7	3.5 – 5.1	mmol/L
Chloride	110	97 – 107	mmol/L
Bicarbonate (HCO₃)	19.0	17 – 27	mmol/L
Creatinine	0.32	0.52 – 0.69	mg/dL
Urea	26	19.26 – 47.29	mg/dL
Calcium	10	8.8 – 10.8	mg/dL
Glucose, Random	98	73 – 112	mg/dL
AST (SGOT)	265	0 – 51	IU/L
ALT (SGPT)	67	0 – 39	IU/L
ALP	433	153 – 367	IU/L
Total Protein	6.6	6.5 – 8.1	g/dL
Total Bilirubin	2.71	0 – 1.2	mg/dL
Albumin	4.7	3.8 – 5.4	g/dL

 Hospital course

The patient deteriorated despite early therapy. On the seventh day of illness, the hemoglobin was 4.8 g/dL and the platelet count was 24×10⁹/L; the blood film showed reactive bone marrow stress, and the viral serologies were negative. The infant was transfused with packed RBCs and platelets to treat the progressive anemia and thrombocytopenia.

**Figure 1:**
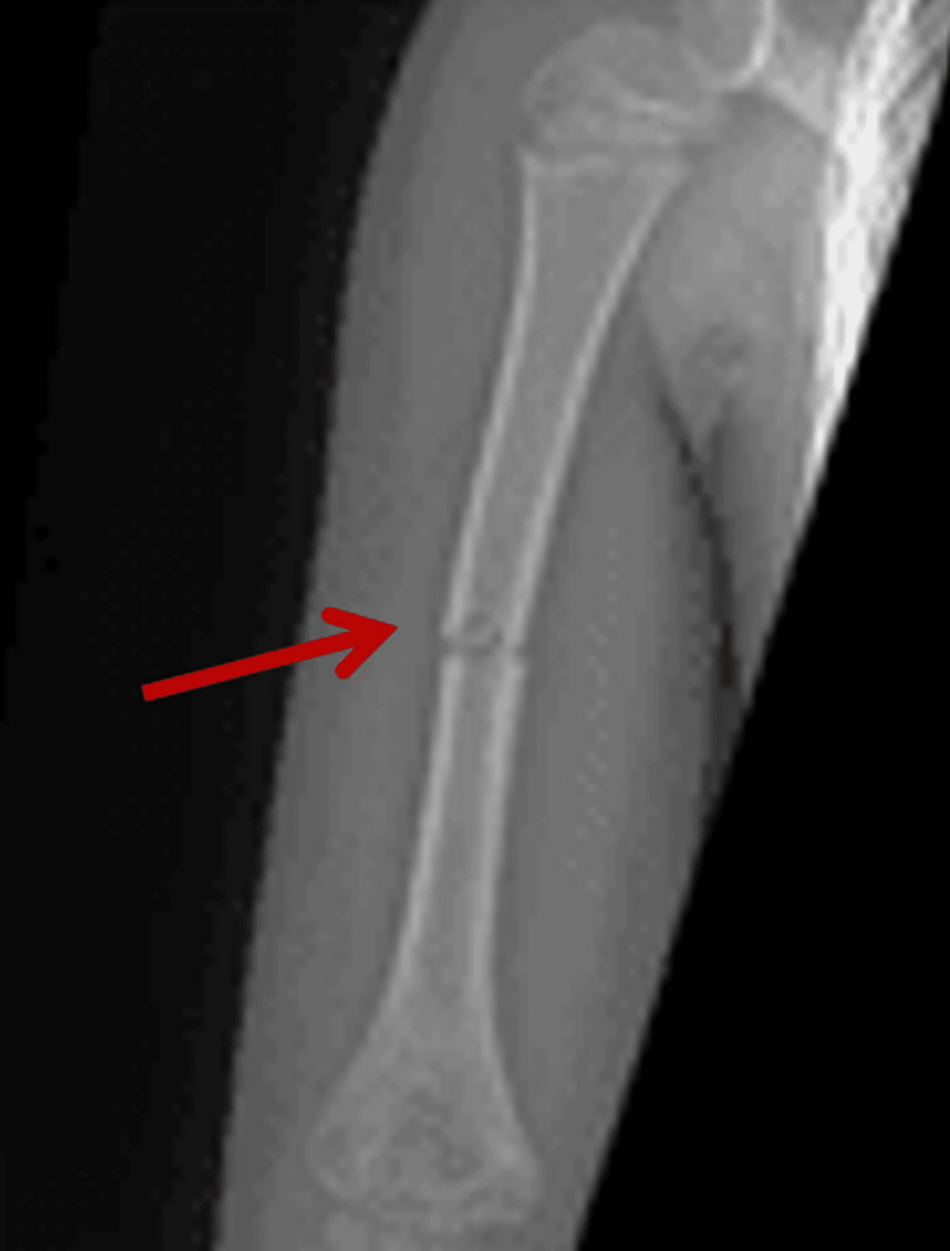
X-ray showing a fracture of the humerus (arrow)

**Figure 2:**
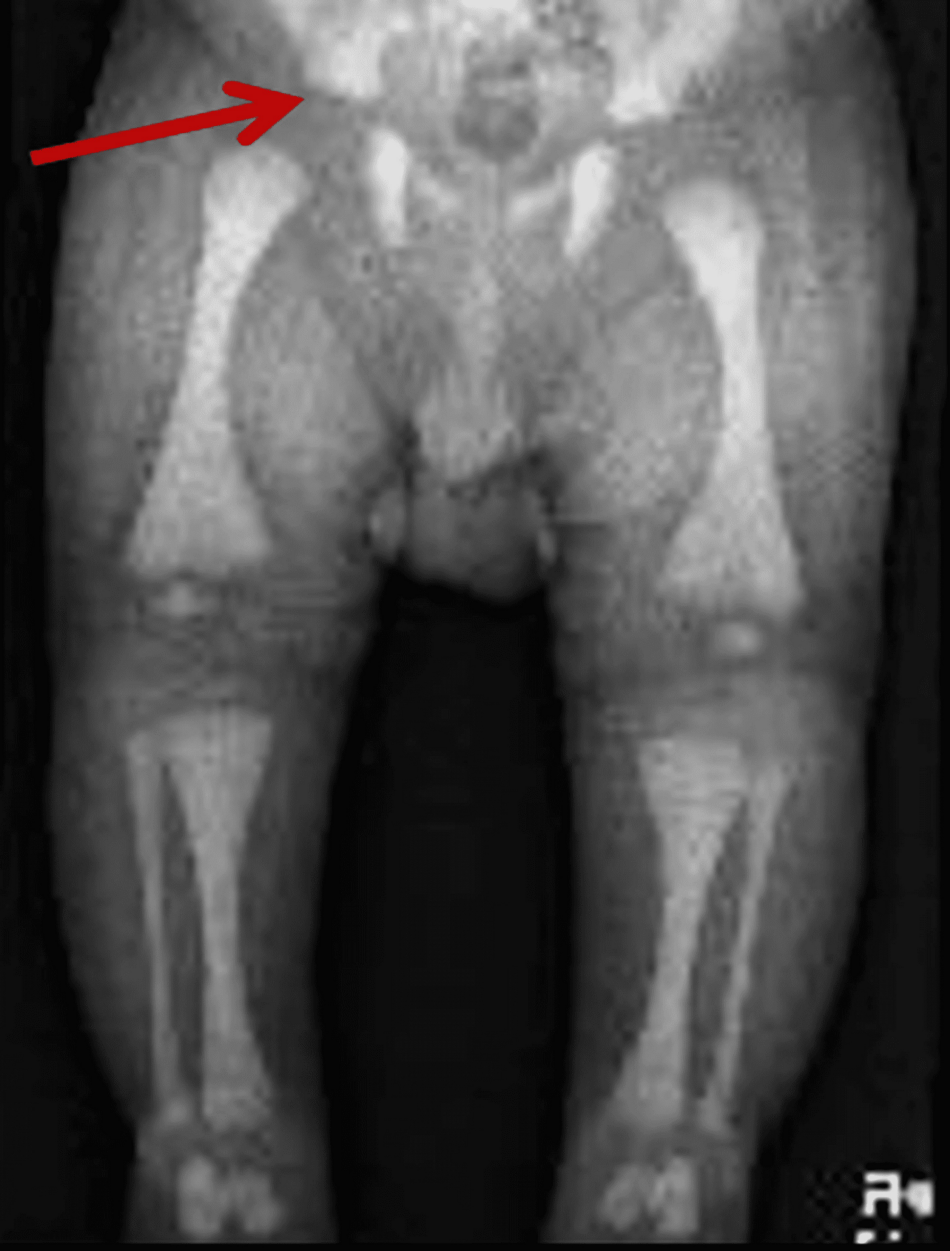
The pubic ramus showing a bone within bone appearance (soft callus)(arrow); long bones show diffuse increased density

On the eighth day of life, the baby presented with a boggy swelling of the right arm; it was painful, and the movement of his arm was limited. Imaging studies demonstrated a humerus fracture (Figure 1), and a skeletal survey was consistent with generalized increased bone density with mild remodelling defects compatible with osteopetrosis. Additional fractures of the bilateral pubic rami were observed as well (Figure 2).

**Figure 3:**
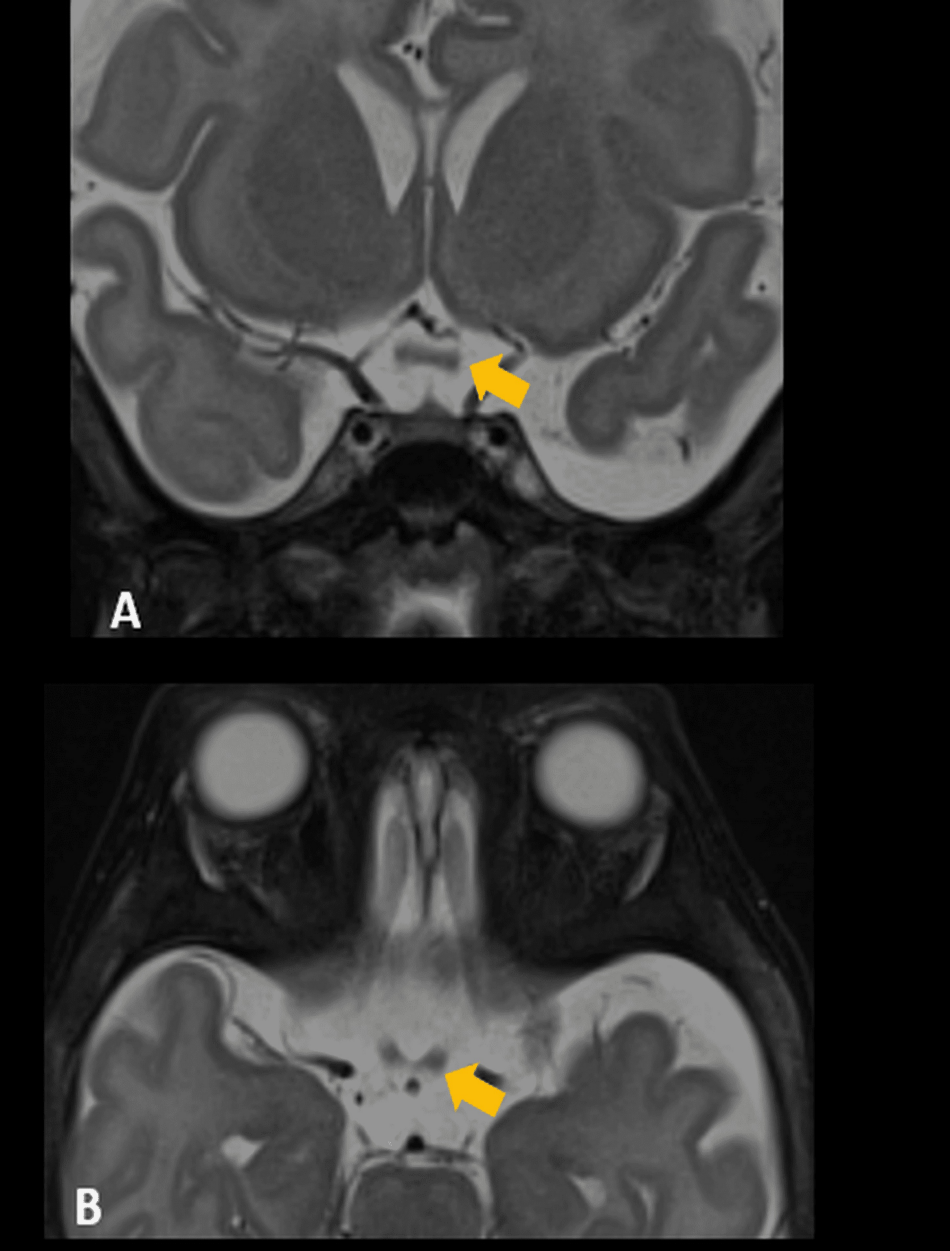
MRI T2 fat saturated sequence in coronal (A) and axial (B) sections shows thinning of the optic chiasm (arrows)

**Figure 4:**
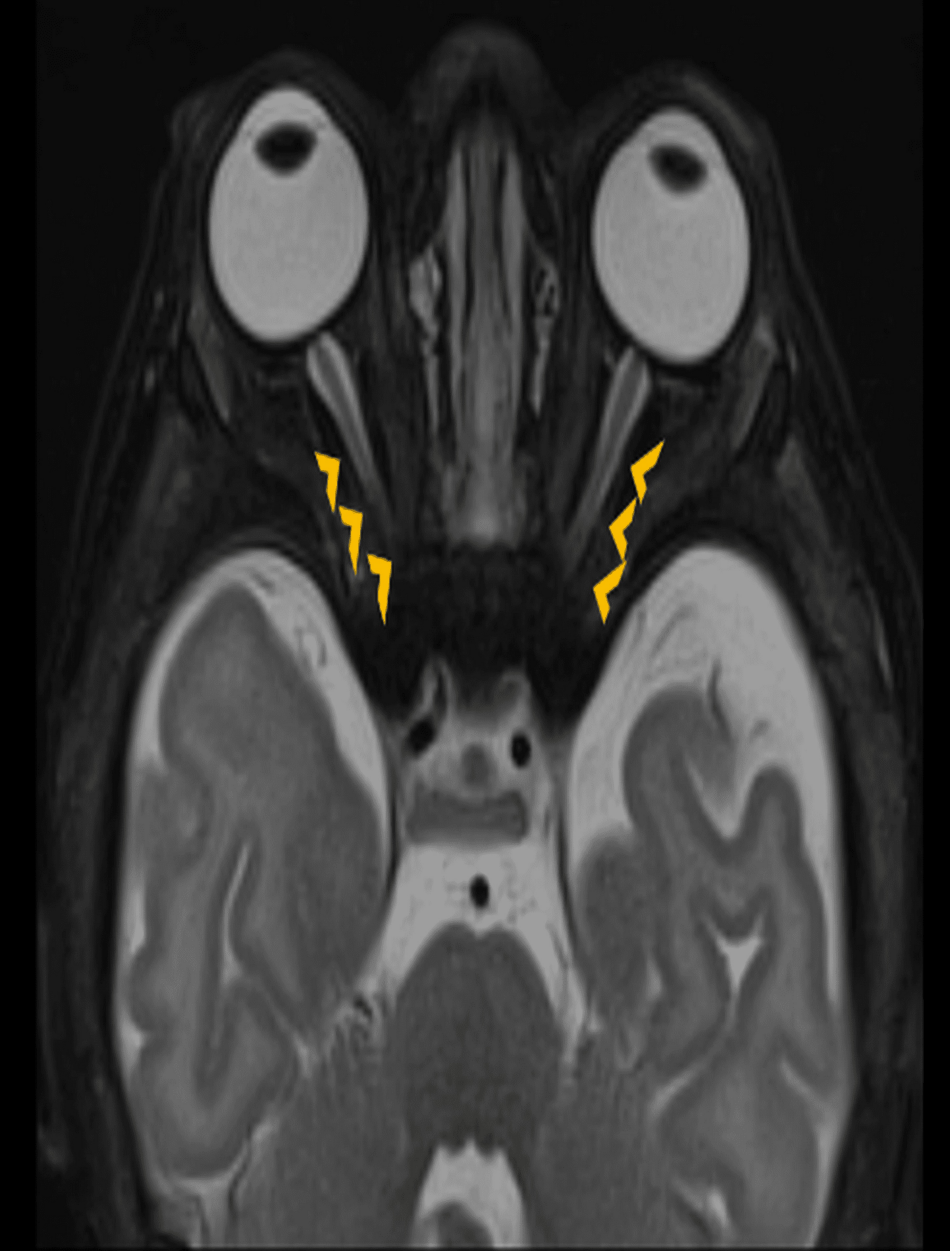
MRI T2 fat saturated sequence in axial section shows thinning of bilateral optic nerves (arrow heads), right more than left

Brain MRI showed cystic hygroma, and the neurosurgical opinion recommended against surgery as long as the patient remained symptomatic. Ophthalmoscopic examination showed bilateral pale optic discs with "salt-and-pepper" appearance, hypoplastic macula, and bilateral absent visual evoked potentials. MRI orbit showed bilateral optic nerve and chiasm thinning more on the right (Figures 3, 4).

Bone metabolism study was suggestive of high parathyroid hormone (PTH) (308 pg/mL), ALP (3781 IU/L), vitamin D insufficiency (17 ng/mL), phosphate (3.46 mg/dL), and normal calcium (8.6 mg/dL). The patient was started on empiric multivitamins and vitamin D3 supplementation at a dose of 800 IU daily. 

Fracture repair was delayed, and radiography demonstrated new bone density and angulation at fracture sites. The patient had also been experiencing difficulty with feeding and greenish vomiting. Upper GI study showed incomplete para-axial malrotation necessitating surgical correction and feeding gastrostomy. Weight gain remained suboptimal despite these interventions.

There was further deterioration of the patient, who developed dystonia, which was treated with chloral hydrate, clonidine, and baclofen. Whole-exome sequencing identified malignant infantile osteopetrosis (type 5) with a homozygous *OSTM1* deleted gene. Regrettably, this type of osteopetrosis is complicated by severe neurologic manifestations and has very few therapies. Bone marrow transplantation was arranged to be performed outside; however, the patient passed away before the operation could be carried out.

## Discussion

Osteopetrosis, commonly and most recognizably known as “marble bone disease” because of the X-ray appearance of the uniformly dense bone, involves a variety of rare genetic bone diseases. The most severe form is malignant infantile osteopetrosis, which affects infants early in life [1,2].

The diagnosis is typically delayed because of the lack of specificity of initial signs like anemia, failure to thrive (FTT), and irritability. The presence of cytopenias and unexplained fractures in this case led to further investigation. Skeletal imaging demonstrated typical characteristics of osteopetrosis, such as generalized bone sclerosis and defects in bone remodeling. Typical features such as “bone-in-bone” appearance and sandwich vertebrae sign coexisted with atypical presentations such as bilateral pubic rami fractures and delayed fracture healing, as also previously described in reports [1,4].

Of particular importance, brain MRI demonstrated a cystic hygroma, which is a rare yet characteristic feature of multisystemic disease for lethal infantile osteopetrosis. Optic disc atrophy and absence of visual evoked potentials were additional ophthalmologic manifestations of neurological injury. These results are in agreement with the complications seen in patients carrying mutations in the *OSTM1* gene (for phenotype associated with a profound neurological defect) [6].

The understanding of a patient's homozygous mutation in the *OSTM1* gene was crucial for prognostic and treatment considerations about this patient. In contrast to other gene defects described in osteopetrosis (*TCIRG1*, *CLCN7*), *OSTM1* mutations are typically not curable by a curative hematopoietic stem cell transplantation because of their early and irreversible neurological involvement [2,6,7]. Accordingly, although no definitive treatment is currently available for some types of malignant infantile osteopetrosis other than hematopoietic stem cell transplantation, characterization and timely genetic diagnosis are crucial for patient selection and outcome [3,7].

The management of malignant infantile osteopetrosis is extensive and multidisciplinary. In this case, the patient needed extensive supportive care, including multiple blood and platelet transfusions, orthopedic stabilization of fractures, treatment with high-frequency vitamin D supplementation, enteric support, surgical intervention to reduce intestinal volvulus, and medications to address dystonia. In spite of these efforts, the clinical course continued to be relentlessly progressive, which is characteristic of the natural history of *OSTM1*-related osteopetrosis [5,6].

Neurological complications, including cranial nerve entrapment and dystonia, are particularly complex. In our patient, dystonia could be controlled with medication; however, the severity of optic nerve involvement suggested irreversible damage to the central nervous system. Literature highlights the importance of timely diagnosis and referral, because once delayed, intervention, especially in advanced neurological stages, reduces treatment options for any potential therapy, including haematopoietic or stem cell transplantation [3,7].

In areas with high rates of consanguinity as the Arabian Peninsula, autosomal recessive diseases, for example, malignant infantile osteopetrosis, have an increased incidence [4]. This case also underscores the necessity of more widespread genetic counseling, carrier testing, and potentially newborn screening in high-risk populations. This could help early detection and intervention in a class of diseases where therapeutic windows are notoriously narrow.

In summary, nefarious multisystemic effects of malignant infantile osteopetrosis, especially those with *OSTM1* mutations, are illustrated in this case. It underscores the value of early radiologic and genetic diagnosis, a coordinated, multidisciplinary care model, and pressing therapeutic advancements to manage subtypes that are not candidates for hematopoietic stem cell transplantation.

## Conclusions

Malignant infantile osteopetrosis is a rare and fatal disorder of infancy, characterized by different clinical manifestations that can hinder early diagnosis. Prenatal diagnosis is largely based on X-ray examination and genetic analysis. While hematopoietic stem cell transplantation may present as a potential cure in some genetic subtypes, the outcome remains grim in most cases with profound neurological involvement as seen in *OSTM1* mutations. Given the autosomal recessive nature of the disease and high morbidity, antenatal genetic counseling and targeted screening for future pregnancies should be offered to affected families. This case report highlights the importance of a multidisciplinary team in treating patients with this devastating disorder and emphasizes the need for further study of novel therapeutic options. 
